# Optimized Tail Bounds for Random Matrix Series

**DOI:** 10.3390/e26080633

**Published:** 2024-07-26

**Authors:** Xianjie Gao, Mingliang Zhang, Jinming Luo

**Affiliations:** 1 Department of Basic Sciences, Shanxi Agricultural University, Jinzhong 030801, China; 2 School of Mathematics and Statistics, Qilu University of Technology (Shandong Academy of Sciences), Jinan 250353, China; zhangml@qlu.edu.cn; 3 School of Mathematical Sciences, Dalian University of Technology, Dalian 116024, China; luojinming@mail.dlut.edu.cn

**Keywords:** tail bound, intrinsic dimension, random matrix series, expectation bound

## Abstract

Random matrix series are a significant component of random matrix theory, offering rich theoretical content and broad application prospects. In this paper, we propose modified versions of tail bounds for random matrix series, including matrix Gaussian (or Rademacher) and sub-Gaussian and infinitely divisible (i.d.) series. Unlike present studies, our results depend on the intrinsic dimension instead of ambient dimension. In some cases, the intrinsic dimension is much smaller than ambient dimension, which makes the modified versions suitable for high-dimensional or infinite-dimensional setting possible. In addition, we obtain the expectation bounds for random matrix series based on the intrinsic dimension.

## 1. Introduction

Random matrix theory is a significant branch of mathematics, which delves into the properties and behavior of random matrices. Its applications span across various fields, including wireless communications [1], combinatorial optimization [2], matrix low-rank approximation [3], neural networks [4,5], and deep learning [6]. Random matrices have a wide range of applications in physics, entropy, and information science. They can provide comprehensive descriptions and analyses when dealing with multiple interacting elements, high-dimensional systems, and complex statistical relationships. Random matrices can better capture the complex interactions between multiple particles or multiple physical processes. When dealing with high-dimensional physical systems with a large number of degrees of freedom, random matrices provide a more natural and effective representation [7,8]. Random matrices can be used to calculate the entropy of complex systems to measure the degree of chaos and uncertainty of the system [9,10]. In the field of information science, random matrices can be used for performance optimization and signal processing of communication systems [11]. Random matrix theory provides a powerful theoretical basis for dealing with problems in these fields. Among them, the random matrix series is an important research topic in the field of random matrix theory, and has wide application and research value.

The study of random matrix theory comprises two branches: asymptotic theory and non-asymptotic theory. There have been several notable asymptotic results in random matrix theory, including Wigner’s semicircle law [12], the Marchenko–Pastur law [13], and the Bai–Yin law [14]. While these asymptotic statements can offer precise limiting results as the matrix dimension approaches infinity, they do not specify the rate at which these probability terms converge to their limits. In response to this challenge, non-asymptotic approaches to analyzing these probability terms have emerged.

Ahlswede and Winter [15] illustrated the application of the Golden–Thompson inequality [16,17] in extending the Laplace transform method to the matrix scenario to derive tail bounds for sums of random matrices. Tropp [18] utilized a corollary of Lieb’s theorem [19] to achieve a significant improvement over the Ahlswede–Winter outcome. To address the notable limitation of results being dependent on the intrinsic dimensions of the matrix, where the bounds become excessively loose in scenarios involving high-dimensional matrices, Hsu et al. [20] presented a tighter analogy to matrix Bernstein’s inequality. Minsker [21] extended Bernstein’s concentration inequalities for random matrices by enhancing the results in [20] through the introduction of the concept of effective rank. Zhang et al. [22] introduced dimension-free tail bounds for the largest singular value of sums of random matrices.

The matrix series form $\sum_{k}x_{k}A_{k}$ has played a crucial role in recent studies [23,24,25], where $x_{k}$ represents a random variable and $A_{k}$ is a fixed matrix. The variable $x_{k}$ can encompass various types of random variables, including Gaussian, Bernoulli, infinitely divisible random variables, and more. Tropp [18] utilized Gaussian series to study the key characteristics of matrix tail bounds. Zhang et al. [26] studied the tail inequalities of the largest eigenvalue of a matrix infinitely divisible (i.d.) series and applied them to optimization problems and compressed sensing.

### 1.1. Related Works

Consider the sum $\sum_{k=1}^{n}\alpha_{k}a_{k}$, where $a_{1},a_{2},\cdots ,a_{n}$ are real numbers and $\alpha_{1},\alpha_{2},\cdots ,\alpha_{n}$ are independent standard Gaussian variables. There is the probability inequality

(1) $$P\left\{\sum_{k=1}^{n}\alpha_{k}a_{k}\geq \sqrt{2\delta^{2}t}\right\}\leq e^{-t}\;\;\;where\;\;\delta^{2}:=\sum_{k=1}^{n}a_{k}^{2}.$$

Let ${\left\{A_{k}\right\}}_{k=1}^{n}$ be a finite sequence of fixed Hermitian matrices with dimension *d*. Tropp [18] gave the following result for any t≥0:

(2) $$P\left\{\lambda_{\max}\left(\sum_{k}\alpha_{k}A_{k}\right)\geq \sqrt{2\eta^{2}t}\right\}\leq d\cdot e^{-t}\;\;\;where\;\;\eta^{2}:=∥\sum_{k}A_{k}^{2}∥.$$

A significant distinction between (1) and (2) is the presence of the matrix dimension factor *d* in the latter. Hsu et al. [20] obtained the following tail bound:

(3) $$P\left\{\lambda_{\max}\left(\sum_{k}\alpha_{k}A_{k}\right)\geq \sqrt{2\eta^{2}t}\right\}\leq \frac{\mathrm{tr}(\Xi )}{\lambda_{\max}\left(\Xi \right)}\cdot \frac{t}{e^{t}-t-1}\;\;\;where\;\;\Xi =\sum_{k=1}^{n}A_{k}^{2}.$$

We observe that the right side of the inequality is the product of two terms. When both terms are smaller, the result will be tighter. Compared with (2), we know that $\mathrm{tr}\left(\Xi \right)/\lambda_{\max}\left(\Xi \right)\leq d$ but $t{\left(e^{t}-t-1\right)}^{-1}>e^{-t}$ for t>0. That is, one term of the results (2) and (3) becomes smaller, while the other term becomes larger. In other words, both outcomes have their respective limitations.

Let ${\left\{\beta_{k}\right\}}_{k=1}^{n}$ be a finite sequence of independent sub-Gaussian random variables. The tail bound can be obtained from

(4) $$P\left\{\lambda_{\max}\left(\sum_{k}\beta_{k}A_{k}\right)\geq t\right\}\leq d\cdot e^{-t^{2}/\left(4c^{2}\eta^{2}\right)}.$$

where *c* is an absolute constant.

Let ${\left\{\gamma_{k}\right\}}_{k=1}^{n}$ be a finite sequence of independent infinitely divisible random variables. Let $B_{1},\cdots ,B_{n}$ be fixed d-dimensional Hermitian matrices with $\lambda_{\max}\left(B_{k}\right)\leq 1$, k=1,⋯,n. For any $0<t<\rho h(M^{-})$, Zhang et al. [26] deduced the following results:

(5) $$\begin{array}{cc} \; & P\left\{\lambda_{\max}\left(\sum_{k}\gamma_{k}B_{k}\right)>t\right\}\\ \; & \leq d\exp\left(-\rho \cdot \int_{0}^{t/\rho }h^{-1}\left(s\right)ds\right), \end{array}$$

where $h\left(M^{-}\right):=\lim_{s↑M}h\left(s\right)$ and $h^{-1}\left(s\right)$ is the inverse of h(s). For any $t\geq \rho h(M^{-})$,

(6) $$P\left\{\lambda_{\max}\left(\sum_{k}\gamma_{k}B_{k}\right)>t\right\}\leq d\exp\left(\rho \phi (M)-Mt\right),$$

where $\rho :=\lambda_{\max}\left(\sum_{k=1}^{K}B_{k}^{2}\right)$.

In addition, Tropp [27] gave the expectation bound for the matrix Gaussian series,

(7) $$E\lambda_{\max}\left(\sum_{k}\alpha_{k}A_{k}\right)\leq \sqrt{2\eta^{2}\log d}.$$

and Zhang et al. [26] also proposed an expectation bound for infinitely divisible matrix series under some given conditions.

However, the significant drawback of the above results lies in the reliance on the ambient dimension of the matrix. The bounds tend to very loose when the matrices have a high dimension. To solve this problem, we optimize the existing theory. Tighter tail bounds for random matrices mean more precise and reliable probability estimates, which enables people to have a more accurate grasp of the behavior of random matrices and helps to improve the accuracy, efficiency and reliability of theory and application.

### 1.2. Overview of Main Results

With the aim of enhancing the limitations of the existing theory and to complement and refine the existing random matrix theory, we put forward optimized tail and expectation bounds for random matrix series in this paper, including matrix Gaussian (or Rademacher), sub-Gaussian, and infinitely divisible (i.d.) series. This makes the modified version potentially adaptable to high-dimensional or infinite-dimensional matrix settings. Taking the matrix Gaussian series as an example, we obtain the tighter conclusion:

(8) $$P\left\{\lambda_{\max}\left(\sum_{k}\alpha_{k}A_{k}\right)\geq \sqrt{2\omega^{2}t}\right\}\leq 2\tilde{d}\cdot e^{-t}\;\;for\;\;t>\omega ,$$

and

(9) $$E\lambda_{\max}\left(\sum_{k}\alpha_{k}A_{k}\right)\leq \sqrt{2}\omega \left(\sqrt{2}+\sqrt{\log(1+\tilde{d})}\right)$$

The $\tilde{d}$ and $\omega^{2}$ will be introduced in detail later in the paper.

The rest of this paper is organized as follows. Section 2 introduces some preliminary knowledge on the intrinsic dimension and Gaussian (or Rademacher), sub-Gaussian, and infinitely divisible (i.d.) distributions. Section 3 gives tail and expectation bounds based on the intrinsic dimension bounds for Gaussian (or Rademacher), sub-Gaussian, and infinitely divisible (i.d.) matrix series. The last section concludes the paper.

## 2. Notations and Preliminaries

In this section, some preliminary knowledge will be provided about the intrinsic dimension of the matrix, and also about Gaussian (or Rademacher), sub-Gaussian, infinitely divisible distributions, and matrix series.

### 2.1. The Intrinsic Dimension

Existing tail bounds on random matrix series depend on the ambient dimension of the matrix. We introduce the concept of the intrinsic dimension, which is much smaller than the ambient dimension in some cases (see also [27]).

**Definition** **1.** *For a positive-semidefinite matrix S, the intrinsic dimension is defined as*

$$\mathrm{intdim}\left(S\right)=\frac{\mathrm{tr}S}{∥S∥}.$$

It can be seen from the definition that the intrinsic dimensions do not significantly affected by changes in the size of the matrix. Actually, when the eigenvalues of S decrease very powerfully, the intrinsic dimension is much smaller than the ambient dimension.

### 2.2. Several Distributions

In this section, we briefly introduce three random distributions and their moment generating functions, including Gaussian (or Rademacher), sub-Gaussian, and infinitely divisible (i.d.) distributions.

The Gaussian distribution is a very important continuous distribution in probability theory and statistics, and is often used to represent real-valued random variables with unknown distribution. Given a Gaussian variable α, the moment generating function (mgf) is given by

(10) $$E\;e^{\theta \alpha }=e^{\theta^{2}/2},\;\;\;\forall \theta \in R.$$

The Rademacher distribution is a discrete probability distribution in which the random variable takes on the value of 1 or −1 with probability $\frac{1}{2}$. Given a Rademacher variable ξ, the moment generating function is given by

(11) $$E\;e^{\theta \xi }\leq e^{\theta^{2}/2},\;\;\;\forall \theta \in R.$$

The sub-Gaussian distribution has strong tail decay, including many distributions, such as uniform and all bounded random distributions. Given a central sub-Gaussian random variable β, it holds that

(12) $$E\;e^{\theta \beta }\leq e^{c^{2}\theta^{2}},\;\;\;\forall \theta \in R$$

where *c* is an absolute constant.

Infinitely divisible (i.d.) distributions are referring to a large class of probability distributions that play an important role in probability theory with limit theorems. A random variable γ has an i.d. distribution if, for any $n\in N_{+}$, there exists independent and identically distributed (i.i.d.) random variables $\gamma_{1},\cdots ,\gamma_{n}$, such that γ has the same distribution as $\gamma_{1}+\cdots +\gamma_{n}$.

In discrete distributions, infinitely divisible distributions include Poisson distribution, negative binomial distribution, and geometric distribution. Among the continuous distributions, Cauchy distribution, Lévy distribution, stable distribution and Gamma distribution are examples of infinitely divisible distributions.

A real-valued random variable γ is i.d. if and only if there exists a triplet $\left(b,\sigma^{2},\nu \right)$, where the characteristic function of γ is defined by

(13) $$E\left\{e^{i\theta \gamma }\right\}=\exp\left(ib\theta -\frac{\sigma^{2}\theta^{2}}{2}+\int_{R}\left(e^{i\theta u}-1-i\theta u1_{|u|<1}\right)\nu \left(du\right)\right),\;\;\;\forall \theta \in R$$

where b∈R, σ≥0 and ν is a Lévy measure. This necessary and sufficient condition is Lévy–Khintchine Theorem.

Let γ be an i.d. random variable with the triplet $\left(b,\sigma^{2},\nu \right)$, and suppose that Eγ=0. Let $M:=\sup\{\theta \geq 0:\;E\left\{e^{\theta |\gamma |}\right\}<+\infty \}$. For any λ≤1 and 0<θ<M,

(14) $$\begin{array}{c} Ee^{\lambda \theta \gamma }\leq \exp\left(\frac{\sigma^{2}\theta^{2}\lambda^{2}}{2}+\lambda^{2}\int_{R}\left(e^{\theta |u|}-\theta \left|u\right|-1\right)\nu \left(du\right)\right). \end{array}$$

The proof can be referred to in [26].

### 2.3. Random Matrix Series

Given *n* fixed matrices $A_{1},A_{2},\cdots ,A_{n}$, a random matrix series is represented as $\sum_{k=1}^{n}x_{k}A_{k}$, where $x_{1},x_{2},\cdots ,x_{n}$ are independent variables. The tail and expectation bounds for random matrix series can be bounded to $P\left\{\lambda_{\max}\left(\sum_{k}x_{k}A_{k}\right)\geq t\right\}$ and $E\lambda_{\max}\left(\sum_{k}x_{k}A_{k}\right).$

## 3. Intrinsic Dimension Bounds for Matrix Series

In this section, we present tail bounds for random matrix series based on intrinsic dimension bounds, and also obtain the expectation bounds.

### 3.1. Matrix Gaussian (or Rademacher) Series with Intrinsic Dimension

This section presents the tail and expectation bounds for matrix Gaussian (or Rademacher) series with an intrinsic dimension.

**Theorem** **1.** *Consider a finite sequence $\left\{A_{k}:k=1,...,n\right\}$ of fixed Hermitian matrices with the same dimensional d, with $\left\{\alpha_{k}\right\}$ being a finite sequence of independent Gaussian (or Rademacher) variables. Introduce the matrix $M⪰\sum_{k}A_{k}^{2}$. Define the following parameters:*

$$\tilde{d}=\mathrm{intdim}\left(M\right)\;\;\;and\;\;\;\omega^{2}=∥M∥$$

*Then, it holds that*

(15) $$P\left\{\lambda_{\max}\left(\sum_{k}\alpha_{k}A_{k}\right)\geq t\right\}\leq 2\tilde{d}\cdot e^{-t^{2}/\left(2\omega^{2}\right)}\;\;for\;\;t>\omega .$$

Compared with the previous results in (2) and (3), our result in (15) improves upon their respective shortcomings, and is more tight. Therefore, our bound is more applicable for the case of high-dimensional matrices.

**Theorem** **2.** *Given a matrix Gaussian (or Rademacher) series $\sum_{k}\alpha_{k}A_{k}$, then it holds that*

(16) $$E\lambda_{\max}\left(\sum_{k}\alpha_{k}A_{k}\right)\leq \sqrt{2}\omega \left(\sqrt{2}+\sqrt{\log(1+\tilde{d})}\right)$$

Compared with the previous result in (7), our result in (16) depends on the intrinsic dimensions of the matrix, and is more applicable for the case of high-dimensional matrices.

The proofs of Theorems 1 and 2 are similar to the proofs of sub-Gaussian matrix series; we omit them here.

### 3.2. Matrix Sub-Gaussian Series with Intrinsic Dimension

This section presents the tail and expectation bounds for matrix sub-Gaussian series with an intrinsic dimension.

**Theorem** **3.** *Consider a finite sequence $\left\{A_{k}:k=1,...,n\right\}$ of fixed Hermitian matrices with the same dimensional d, with $\left\{\beta_{k}\right\}$ being a finite sequence of independent central sub-Gaussian variables. Introduce the matrix $M⪰\sum_{k}A_{k}^{2}$. Define the following parameters:*

$$\tilde{d}=\mathrm{intdim}\left(M\right)\;\;\;and\;\;\;\omega^{2}=∥M∥$$

*Then, it holds that*

(17) $$P\left\{\lambda_{\max}\left(\sum_{k}\beta_{k}A_{k}\right)\geq t\right\}\leq 2\tilde{d}\cdot e^{-t^{2}/\left(4c^{2}\omega^{2}\right)}\;\;for\;\;t>\sqrt{2}c\omega .$$

*where c is an absolute constant.*

Before proving this theorem, we first introduce a proposition [27] that will be used in the proof process. This proposition is a key step in our proof.

**Proposition** **1.** *Let Y be a random Hermitian matrix. Let $\psi :R\to R_{+}$ be a nonnegative function that is nondecreasing on [0,∞). For each t≥0,*

(18) $$P\left\{\lambda_{\max}\left(Y\right)\geq t\right\}\leq \frac{1}{\psi (t)}E\;\mathrm{tr}\psi \left(Y\right).$$

**Proof.**  Let the sum

$$Y=\sum_{k}\beta_{k}A_{k}.$$

Fix a number θ>0, and define the function $\psi \left(t\right)=\max\left\{0,\;e^{\theta t}-1\right\}$ for t∈R. For t≥0, Proposition 1 states that

(19) $$P\left\{\lambda_{\max}\left(Y\right)\geq t\right\}\leq \frac{1}{\psi (t)}E\;\mathrm{tr}\psi \left(Y\right)=\frac{1}{e^{\theta t}-1}E\;\mathrm{tr}\left(e^{\theta Y}-I\right).$$

Introduce the matrix $M⪰\sum_{k}A_{k}^{2}$. According to the mgf of a sub-Gaussian random variable in (12) and the transfer rule (consider a real-valued function *f*, if f(a)≤g(a) for a∈I, then f(A)⪯g(A), when the eigenvalues of A lie in *I*), it can be known that

(20) $$E\;\mathrm{tr}e^{\theta Y}\leq \mathrm{tr}\exp\left(\theta^{2}c^{2}\sum_{k}A_{k}^{2}\right)=\mathrm{tr}\exp\left(g\left(\theta \right)\cdot M\right)\;\;where\;\;g\left(\theta \right)=c^{2}\theta^{2}.$$

Introduce the function $\varphi \left(a\right)=e^{a}-1$, and observe that

$$E\;\mathrm{tr}\left(e^{\theta Y}-I\right)\leq \mathrm{intdim}\left(M\right)\varphi \left(g(\theta )∥M∥\right).$$

Define the following parameters:

$$\tilde{d}=\mathrm{intdim}\left(M\right)\;\;\;and\;\;\;\omega^{2}=∥M∥$$

We have

(21) $$E\;\mathrm{tr}\left(e^{\theta Y}-I\right)\leq \tilde{d}\cdot \varphi \left(g\left(\theta \right)\omega^{2}\right)\leq \tilde{d}\cdot e^{g\left(\theta \right)\cdot \omega^{2}}.$$

Next, combine the bound (21) and the probability bound to obtain

(22) $$P\left\{\lambda_{\max}\left(Y\right)\geq t\right\}\leq \tilde{d}\cdot \frac{e^{\theta t}}{e^{\theta t}-1}\cdot e^{-\theta t+g\left(\theta \right)\cdot \omega^{2}}\leq \tilde{d}\cdot \left(1+\frac{1}{\theta t}\right)\cdot e^{-\theta t+g\left(\theta \right)\cdot \omega^{2}}.$$

We use the following formula to control the fraction:

$$\frac{e^{a}}{e^{a}-1}=1+\frac{1}{e^{a}-1}\leq 1+\frac{1}{a}\;\;for\;a\geq 0.$$

We select $\theta =t/(2c^{2}\omega^{2})$ to obtain

(23) $$P\left\{\lambda_{\max}\left(\sum_{k}\beta_{k}A_{k}\right)\geq t\right\}\leq \tilde{d}\left(1+\frac{2c^{2}\omega^{2}}{t^{2}}\right)e^{-t^{2}/\left(4c^{2}\omega^{2}\right)}$$

Install the assumption that $t>\sqrt{2}c\omega$ and yield the conclusion. □

Since the large deviation inequality considers the case where *t* is large, the limitation $t>\sqrt{2}c\omega$ is reasonable.

**Theorem** **4.** *Given a matrix sub-Gaussian series $\sum_{k}\beta_{k}A_{k}$, then it holds that*

(24) $$E\lambda_{\max}\left(\sum_{k}\beta_{k}A_{k}\right)\leq 2c\omega \left(\sqrt{2}+\sqrt{\log(1+\tilde{d})}\right).$$

**Proof.**  Fix a number $t>\mu >\sqrt{2}c\omega$.

(25) $$\begin{array}{cc} E\lambda_{\max}\left(\sum_{k}\beta_{k}A_{k}\right)= & \int_{0}^{\infty }P\left\{\lambda_{\max}\left(\sum_{k}\beta_{k}A_{k}\right)\geq t\right\}dt\\ \leq & \int_{0}^{\mu }P\left\{\lambda_{\max}\left(\sum_{k}\beta_{k}A_{k}\right)\geq t\right\}dt+2\tilde{d}\int_{\mu }^{\infty }e^{-t^{2}/\left(4c^{2}\omega^{2}\right)}dt\\ \leq & \mu +2\tilde{d}\int_{\mu }^{\infty }e^{-t^{2}/\left(4c^{2}\omega^{2}\right)}dt\\ \leq & \mu +2\tilde{d}\sqrt{2}c\omega \int_{\mu }^{\infty }\frac{t}{2c^{2}\omega^{2}}e^{-t^{2}/\left(4c^{2}\omega^{2}\right)}dt\\ = & \mu +2\tilde{d}\cdot \sqrt{2}c\omega e^{-\mu^{2}/\left(4c^{2}\omega^{2}\right)} \end{array}$$

Select $\mu =2c\omega \sqrt{\log(1+\tilde{d})}$,

(26) $$\begin{array}{cc} E\lambda_{\max}\left(\sum_{k}\beta_{k}A_{k}\right)\leq & 2c\omega \sqrt{\log(1+\tilde{d})}+2\tilde{d}\cdot \sqrt{2}c\omega e^{-\log(1+\tilde{d})}\\ = & 2c\omega \sqrt{\log(1+\tilde{d})}+\frac{2\tilde{d}}{1+\tilde{d}}\sqrt{2}c\omega \\ \leq & 2c\omega \sqrt{\log(1+\tilde{d})}+2\sqrt{2}c\omega \\ = & 2c\omega \left(\sqrt{2}+\sqrt{\log(1+\tilde{d})}\right) \end{array}$$

□

### 3.3. Matrix Infinite Divisible Series with Intrinsic Dimension

This section presents the tail and expectation bounds for matrix i.d. series with an intrinsic dimension.

**Theorem** **5.** *Consider a finite sequence $\left\{B_{k}:k=1,...,n\right\}$ of fixed Hermitian matrices with the same dimensional d, $\lambda_{\max}\left(B_{k}\right)\leq 1$, with $\left\{\gamma_{k}\right\}$ being a finite sequence of independent centered i.d. with the triplet $\left(b,\sigma^{2},\nu \right)$ variable, such that $Ee^{\theta |\gamma |}<+\infty$ for some θ>0. Introduce the matrix $M⪰\sum_{k}B_{k}^{2}$. Define the following parameters:*

$$\tilde{d}=\mathrm{intdim}\left(M\right)\;\;\;and\;\;\;\omega^{2}=∥M∥$$

*Then, holds that*

(27) $$P\left\{\lambda_{\max}\left(\sum_{k}\gamma_{k}B_{k}\right)\geq t\right\}\leq 2\tilde{d}\cdot \exp\left(-\omega^{2}\cdot \int_{0}^{t/\omega^{2}}h^{-1}\left(s\right)ds\right)\;\;for\;\;h\left(M^{-}\right)\omega^{2}>t>\frac{1}{h^{-1}\left(t/\omega^{2}\right)}.$$

*where $h(M^{-})$ is the left limit at M, with*

$$M:=\sup\{\theta >0:Ee^{\theta |\gamma |}<+\infty \},$$

*and $h^{-1}$ is the inverse of*

$$h\left(s\right)=\sigma^{2}s+\int_{R}\left|u\right|I\left(e^{s|u|}-1\right)\nu \left(du\right),\;\;0<s<M.$$

*For any $t\geq h\left(M^{-}\right)\omega^{2}$, we have*

$$P\left\{\lambda_{\max}\left(\sum_{k}\gamma_{k}B_{k}\right)>t\right\}\leq 2\tilde{d}\cdot \exp\left(\omega^{2}\phi \left(M\right)-Mt\right),$$

*where*

(28) $$\phi \left(\theta \right):=\frac{\sigma^{2}\theta^{2}}{2}+\int_{R}\left(e^{\theta |u|}-\theta \left|u\right|-1\right)\nu \left(du\right).$$

Compared with the previous result in [26], our results depend on the intrinsic dimensions of the matrix and are more applicable for the case of high-dimensional matrices.

**Proof.**  Let the sum

$$Y=\sum_{k}\gamma_{k}B_{k}.$$

Introduce the matrix $M⪰\sum_{k}B_{k}^{2}$. Similar to the above proof, according to the mgf of i.d. random variable in (14) and the transfer rule, we can obtain

(29) $$P\left\{\lambda_{\max}\left(Y\right)\geq t\right\}\leq \tilde{d}\cdot \frac{e^{\theta t}}{e^{\theta t}-1}\cdot e^{-\theta t+\phi \left(\theta \right)\cdot \omega^{2}}\leq \tilde{d}\cdot \left(1+\frac{1}{\theta t}\right)\cdot e^{-\theta t+\phi \left(\theta \right)\cdot \omega^{2}}.$$

Next, we minimize the right-hand side of (29) with respect to θ. Since $Ee^{\theta \gamma }<+\infty$ for all 0<θ<M, ϕ(θ) is infinitely differentiable on (0,M), with

(30) $$\begin{array}{cc} \phi^{\prime }\left(\theta \right) & :=h\left(\theta \right)=\sigma^{2}\theta +\int_{R}\left|u\right|\left(e^{\theta |u|}-1\right)\nu \left(du\right)>0, \end{array}$$

and

(31) $$\begin{array}{c} \phi^{\prime \prime }\left(\theta \right)=\sigma^{2}+\int_{R}{\left|u\right|}^{2}e^{\theta |u|}\nu \left(du\right)>0. \end{array}$$

Since $\phi \left(0\right)=h\left(0\right)=h^{-1}\left(0\right)=0$, we have

(32) $$\begin{array}{cc} \phi \left(h^{-1}\left(t/\omega^{2}\right)\right)= & \int_{0}^{h^{-1}\left(t/\omega^{2}\right)}h\left(s\right)\;ds\\ = & \int_{0}^{t/\omega^{2}}s\;dh^{-1}\left(s\right)\\ = & \left(t/\omega^{2}\right)\cdot h^{-1}\left(t/\omega^{2}\right)-\int_{0}^{t/\omega^{2}}h^{-1}\left(s\right)\;ds. \end{array}$$

We select $\theta =h^{-1}\left(t/\omega^{2}\right)$ to obtain

$$\begin{array}{cc} \min_{0<\theta <M}\left\{\omega^{2}\cdot \phi \left(\theta \right)-\theta \cdot t\right\}= & \omega^{2}\cdot \phi \left(h^{-1}\left(t/\omega^{2}\right)\right)-t\cdot h^{-1}\left(t/\omega^{2}\right)\\ = & -\omega^{2}\cdot \int_{0}^{t/\omega^{2}}h^{-1}\left(s\right)ds. \end{array}$$

Install the assumption that $t>\frac{1}{h^{-1}\left(t/\omega^{2}\right)}$; we have

$$P\left\{\lambda_{\max}\left(\sum_{k}\gamma_{k}B_{k}\right)\geq t\right\}\leq 2\tilde{d}\cdot \exp\left(-\omega^{2}\cdot \int_{0}^{t/\omega^{2}}h^{-1}\left(s\right)ds\right).$$

Actually, when $t\geq h\left(M^{-}\right)\omega^{2}$, according to the convexity of $\omega^{2}\phi \left(\theta \right)-\theta t$ with respect to θ>0 and the monotonicity of $h^{-1}\left(s\right)$ (s>0), the solution to the optimization problem is θ=M. Thus, for any $t\geq h\left(M^{-}\right)\omega^{2}$, we have

$$P\left\{\lambda_{\max}\left(\sum_{k}\gamma_{k}B_{k}\right)>t\right\}\leq 2\tilde{d}\cdot \exp\left(\omega^{2}\phi \left(M\right)-Mt\right),$$

□

Given some specific settings of the measure ν, we can obtain the following corollary.

**Corollary** **1.** *Assume ν has a bounded support, i.e., there exists a positive constant a<∞ such that ν((−∞,−a)∪(a,∞))=0 and ν([−a,a])≠0. Let*

(33) R=inf{a>0:ν({u:|u|>a})=0}.

*It follows that R<∞. Then, for any t>0,*

(34) $$\begin{array}{cc} \; & P\left\{\lambda_{\max}\left(\sum_{k}\gamma_{k}B_{k}\right)>t\right\}\\ \leq & 2\tilde{d}\cdot \exp\left(-\frac{\omega^{2}\left(\sigma^{2}+V\right)}{R^{2}}\cdot Q\left(\frac{Rt}{\omega^{2}\left(\sigma^{2}+V\right)}\right)\right), \end{array}$$

*where $V:=\int_{R}{\left|u\right|}^{2}\nu \left(du\right)$, and*

(35) Q(s):=(1+s)·log(1+s)−s.

**Proof.**  Since the support is supp(ν)⊆[−R,R], it holds that $Ee^{\theta |\gamma |}<+\infty$ for any θ>0. Thus, we have

(36) $$\begin{array}{cc} h(\theta )= & \sigma^{2}\theta +\int_{R}\left|u\right|\left(e^{\theta |u|}-1\right)\nu \left(du\right)\\ = & \sigma^{2}\theta +\int_{|u|\leq R}{\left|u\right|}^{2}\left(\sum_{k=1}^{\infty }\frac{\theta^{k}{\left|u\right|}^{k-1}}{k!}\right)\nu \left(du\right)\\ \leq & \sigma^{2}\theta +\int_{|u|\leq R}{\left|u\right|}^{2}\left(\sum_{k=1}^{\infty }\frac{\theta^{k}R^{k-1}}{k!}\right)\nu \left(du\right)\\ = & \sigma^{2}\theta +V\left(\frac{e^{\theta R}-1}{R}\right)\leq \left(\sigma^{2}+V\right)\left(\frac{e^{\theta R}-1}{R}\right). \end{array}$$

Denote $p\left(\theta \right):=\left(\sigma^{2}+V\right)\left(\frac{e^{\theta R}-1}{R}\right)$ with the inverse function $p^{-1}\left(s\right)=\frac{1}{R}\cdot \log\left(1+\frac{Rs}{\sigma^{2}+V}\right)$(s>0). Since h(θ) and p(θ) (θ>0) are strictly increasing functions, their inverse functions satisfy the relation $p^{-1}\left(s\right)\leq h^{-1}\left(s\right)$ for all s>0. By combining (27) and (36), we obtain, for any $t>\frac{1}{h^{-1}\left(t/\omega^{2}\right)}$,

$$\begin{array}{cc} \; & P\left\{\lambda_{\max}\left(\sum_{k}\gamma_{k}B_{k}\right)>t\right\}\\ \leq & 2\tilde{d}\cdot \exp\left(-\omega^{2}\cdot \int_{0}^{t/\omega^{2}}h^{-1}\left(s\right)ds\right)\\ \leq & 2\tilde{d}\cdot \exp\left(-\omega^{2}\cdot \int_{0}^{t/\omega^{2}}\frac{1}{R}\cdot \log\left(1+\frac{Rs}{\sigma^{2}+V}\right)ds\right)\\ = & 2\tilde{d}\cdot \exp\left(-\frac{\omega^{2}\left(\sigma^{2}+V\right)}{R^{2}}\cdot q\left(\frac{Rt}{\omega^{2}\left(\sigma^{2}+V\right)}\right)\right), \end{array}$$

where q(s):=(1+s)·log(1+s)−s. This completes the proof. □

Given a matrix i.d. series $\sum_{k}\gamma_{k}B_{k}$, then it holds that

(37) $$E\lambda_{\max}\left(\sum_{k}\gamma_{k}B_{k}\right)=\int_{0}^{\infty }P\left\{\lambda_{\max}\left(\sum_{k}\gamma_{k}B_{k}\right)\geq t\right\}dt.$$

In other words, in the case where the tail bound is integrable, we can use Formula (37) to obtain the expectation bound based on the intrinsic dimensions for matrix i.d. series.

Compared with existing studies, our results are based on the intrinsic dimension of the matrix. The tail and expectation bounds are tighter than the previous results. Therefore, our bounds are more applicable for the case of high-dimensional matrices.

In addition, by using the Hermitian dilation, our results can also be extended to the scenario of non-Hermitian random matrix series. Consider that the general random matrix series $\sum_{k}x_{k}C_{k}$, $∥\sum_{k}x_{k}C_{k}∥=\lambda_{\max}\left(\sum_{k}x_{k}\varphi \left(C_{k}\right)\right)$ is established, among which

$$\varphi \left(C_{k}\right):=\left(\begin{array}{cc} 0 & C_{k}\\ C_{k}^{*} & 0 \end{array}\right).$$

Thus, we may invoke each theorem to obtain tail and expectation bounds for the norm of the random matrix series.

## 4. Conclusions

In this paper, we propose optimized tail and expectation bounds for random matrix series, including matrix Gaussian (or Rademacher) and sub-Gaussian and infinitely divisible (i.d.) series. Different from existing studies, our results depend on intrinsic dimension rather than ambient dimension, and are more suitable for the case of high-dimensional matrices.

In future work, we will use the obtained results to study tail bounds and expectation bounds for other eigenvalues of random matrix series.

## Data Availability

No new data were created or analyzed in this study. Data sharing is not applicable to this article.
